# A Comprehensive Review of Treatment Plans for Marginal Enamel Fractures in Anterior Teeth

**DOI:** 10.3390/biomimetics9120770

**Published:** 2024-12-18

**Authors:** Riccardo Favero, Alessandro Scattolin, Martina Barone, Giampaolo Drago, Rim Bourgi, Vincenzo Tosco, Riccardo Monterubbianesi, Angelo Putignano

**Affiliations:** 1 Department of Neurosciences, Università degli Studi di Padova, 35122 Padova, Italy; infofavero@gmail.com (R.F.); alessandroscattolin30@gmail.com (A.S.); martinabarone4@gmail.com (M.B.); dr.giampaolo@gmail.com (G.D.); 2 Department of Restorative Dentistry, School of Dentistry, Saint-Joseph University, Beirut 1107 2180, Lebanon; rim.bourgi@net.usj.edu.lb (R.B.);; 3 Department of Clinical Sciences and Stomatology, Università Politecnica delle Marche, 60121 Ancona, Italy; 4 National Institute of Health and Science of Aging (IRCCS INRCA), 60124 Ancona, Italy

**Keywords:** anterior marginal enamel fractures, fractures, restorative dentistry, operative dentistry

## Abstract

Marginal enamel fractures (MEF) are a common clinical concern in dentistry, particularly in anterior teeth. These fractures occur at the enamel margins and their etiopathogenesis involves a complex interplay of mechanical, chemical, and biological factors. The ongoing research focuses on an overview of MEF to improve the knowledge about this condition. Understanding the multifaceted nature of MEF is crucial for devising effective preventive and therapeutic strategies in contemporary restorative dentistry. Indeed, mechanical stresses, such as occlusal forces and parafunctional habits are primary contributors for MEF. Additionally, it can happen at the enamel-restoration interface due to expansion and contraction of restorative materials. Chemical degradation, including acid erosion and the breakdown of adhesive bonds, further exacerbates the vulnerability of enamel. Biological factors, such as enamel composition and the presence of micro-cracks also play a role in the development of MEF. Clinical management of MEF involves subtractive or additive techniques, repairing or replacing the compromised tooth structure using techniques to ensure the integration with the natural enamel.

## 1. Introduction

**Figure 1:**
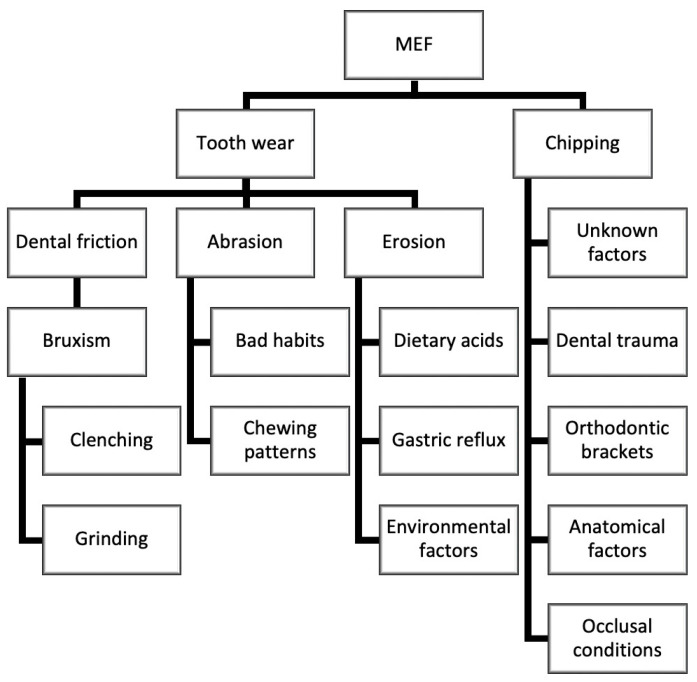
Etiology of MEFs.

Marginal enamel fractures (MEF) at the incisal edge of anterior teeth are a form of uncomplicated dental fracture, characterized by the localized loss of enamel and, potentially, other hard dental tissues. These fractures can result in the detachment of enamel and portions of dentin. MEF can arise from several factors such as tooth wear and chipping (Figure 1).

Tooth wear is defined as the cumulative loss of mineralized tooth substance due to physical or chemical–physical processes unrelated to dental caries. Tooth wear can provoke different conditions: dental friction, which is the physical loss of mineralized tooth substance caused by tooth-to-tooth contact; abrasion, which results from contact with external objects; and erosion, the dissolution of tooth hard tissue by acids [1,2,3]. Dental friction can be caused by bruxism, which can be static (clenching) or dynamic (grinding) and may occur nocturnally (sleep bruxism) or diurnally (awake bruxism) [4,5]. These activities exert substantial forces on tooth surfaces, leading to dental tissue damage. Habits such as holding a pipe between the teeth, chewing on pens, onychophagia, or specific chewing patterns involving the anterior teeth can also cause abrasion [1,6]. On the other hand, erosion, primarily caused by dietary acids, gastric reflux, and environmental factors, leads to the chemical dissolution of tooth hard tissue, thereby increasing the susceptibility to marginal enamel fractures.

**Figure 2:**
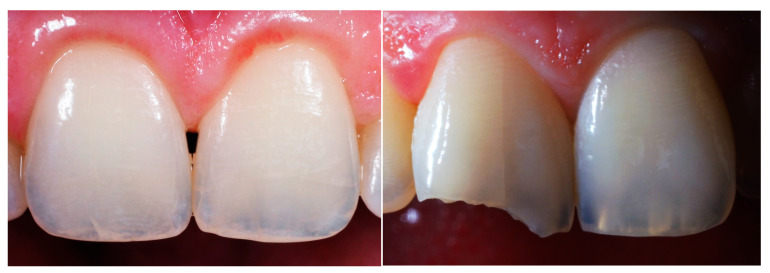
Examples of marginal enamel fractures. Images were modified in color, value and contrast to highlight the fractures.

MEF is often anticipating by chipping, which involves minor fractures of the incisal margin of incisors and canines, leading to the detachment of enamel fragments and possibly dentin [7,8] (Figure 2). Chipping can result from unknown factors or be due to dental trauma, with the maxillary anterior teeth, particularly the central incisors, being the most frequently affected [9]. Anatomical factors, such as Class II skeletal and dental relationships, and increased overjet and overbite, can heighten the risk of trauma to the anterior teeth of the upper dental arch. Orthodontic brackets, particularly ceramic ones, can cause significant contact wear due to their high hardness [10,11]. Enamel fractures can also occur during the debonding phase of orthodontic treatment, affecting either the buccal or lingual surface depending on bracket placement [12,13]. Since MEF mainly involve the anterior teeth, the clinician must solve not only the structural problems, but also the aesthetic demands of the patient. A mimetic and aesthetic treatment is therefore necessary to achieve a good result.

**Figure 3:**
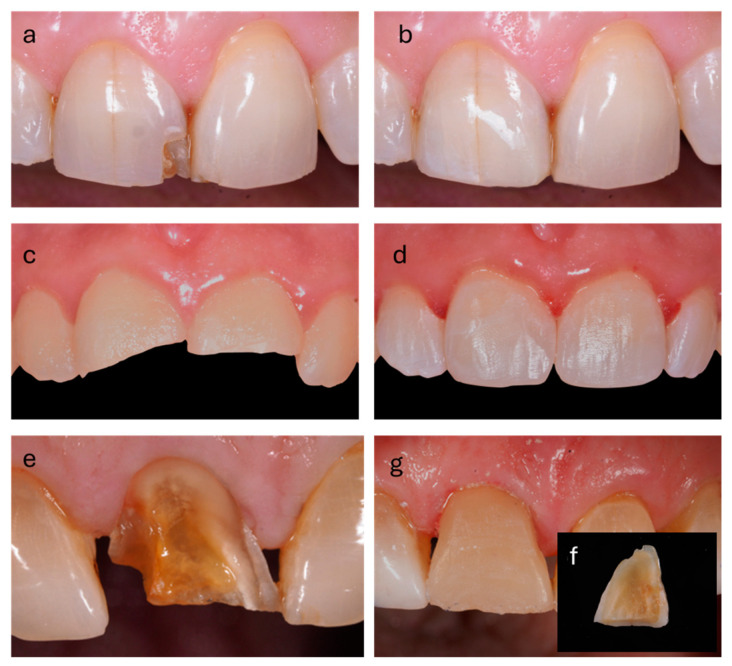
Pictures illustrate the pre- and post-treatment situation of MEF for a mimetic restoration: (**a**,**b**) show the condition before and after direct restoration on 1.1; (**c**,**d**) illustrate the pre- and post-treatment phases of a large enamel fracture involving 1.1 and 2.1; (**e**–**g**) represent an extreme case of enamel fracture with detachment of the buccal layer. The fragment was bonded to the remaining dental tissue.

In this light, several approaches can be considered including cosmetic enamel recontouring (enameloplasty) or the application of direct or indirect restorative materials (Figure 3). The choice of the most appropriate technique requires careful planning that considers dental anatomy, dento-facial harmony, and esthetic proportions [2].

The purpose of this review is to provide an overview of anterior-tooth MEF and the available techniques which will be divided into subtractive and additive techniques.

## 2. Subtractive Technique

The subtractive technique is represented by cosmetic enamel recontouring or the enameloplasty of the tooth. This technique provides the selective removal and contextual remodeling of the hard tissue of the tooth affected by MEFs, without the successive apposition of any restorative material [14]. The amount of marginal enamel to be removed needs to be carefully and precisely defined. To guide the dental tissue removal and to pre-visualize the final result, the areas to be modified can be demarcated with a dedicated dark pen or pencil. The enameloplasty is performed by using diamond bur and stainless-steel abrasive strips. The procedure does not require local anesthesia, also allowing for an indication of the possible degree of sensitivity of the tooth. Then, the surface needs to be finished with a carbide bur on a contra-angle and polished with a coarse silicone cup. 

The subtractive technique is not an elective treatment, but in some cases, it could be indicated. When performed, the criteria of symmetry and minimal invasiveness must be respected. Table 1 and Table 2 describe, respectively, the advantages and disadvantages of the subtractive technique and its indications and contraindications.

The subtractive technique can also be applied for the finishing of an orthodontic case, namely the last phase of the orthodontic treatment in which the clinician acts on the microaesthetics, by modifying factors as tooth morphology, gingival festooning, and alignment on the vertical plane [15]. For example, if at the end of an orthodontic treatment a tooth presents a longer crown than the contralateral one that is proportionately longer than that of the other elements, with possible asymmetry of the gingival festoon, the subtractive ameloplasty of the element-affected MEF can be indicated to achieve a better global aesthetic point of view. Notably, the clinician should limit the subtractive technique to a few limited and selected cases. Indeed, the additive technique should be preferred.

## 3. Additive Technique

Additive treatment concerns adding material to MEF and can be performed by a direct composite reconstruction (direct additive technique) or an indirect restoration (indirect additive technique).

### 3.1. Direct Additive Technique

Direct composite restoration can be performed with various latest-generation composite materials and combined with different additive restoration techniques. The choice of technique should be modulated based on the type of enamel defect.

**Figure 4:**
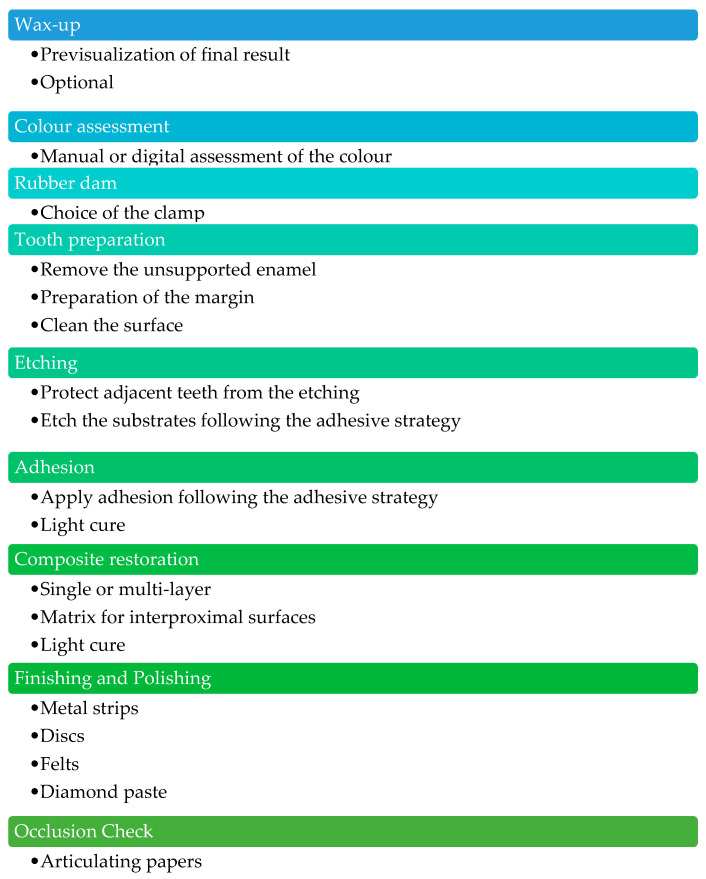
Description of the main steps of direct composite restoration.

Additive treatment with direct composite restoration involves the reconstruction of the incisal margin-affected MEF with minimal preparation, in order to act as conservatively as possible. Figure 4 summarizes the phases of performing a direct composite restoration [16,17,18,19,20].

The design of the preparation is important for the clinical success of the direct restoration as well the restorative technique. An in vitro study published in 2019 [21] evaluated and compared the effect of enamel preparation designs on the fracture resistance of nanocomposites. It emerged that chamfer and stair-step chamfer preparations had a similar resistance to fractures, with better results than the bevel design, due to the greater quantity of composite at the margin. As regards the type of restoration failures, the cohesive type appeared highest in the chamfer and stair-step chamfer designs, and the adhesive failure appeared highest in the bevel design. Ammannato et al. [17] in 2018 suggested a “no-preparation” technique for the enamel; this involved only cleaning with pumice and chlorhexidine and sandblasting with alumina oxide 50 μm.

Table 3 describes the advantages and disadvantages of the additive technique with direct composite restoration, while in Table 4, we present the indications and contraindications of this technique.

In general, a minimal preparation of the enamel before proceeding with the direct composite reconstruction of the incisal margin is sufficient. However, a more aggressive preparation might be needed if there are MEF that could compromise the stability and aesthetics of the reconstruction [22,23]. In fact, deeply undermined fractured margins could favor the detachment of the restoration, as well as the breaking of the margin itself. So, it appears very important to homogenize the structure of the incisal margin before its reconstruction with composite material. Although it is outside of the goal of this paper, clinicians should consider both the preparation of the margin and the restorative technique [24].

### 3.2. Indirect Additive Technique

Indirect restoration can be performed with composite or ceramic veneers, which are applied on the vestibular surface of the tooth crown after a minor preparation.

**Figure 5:**
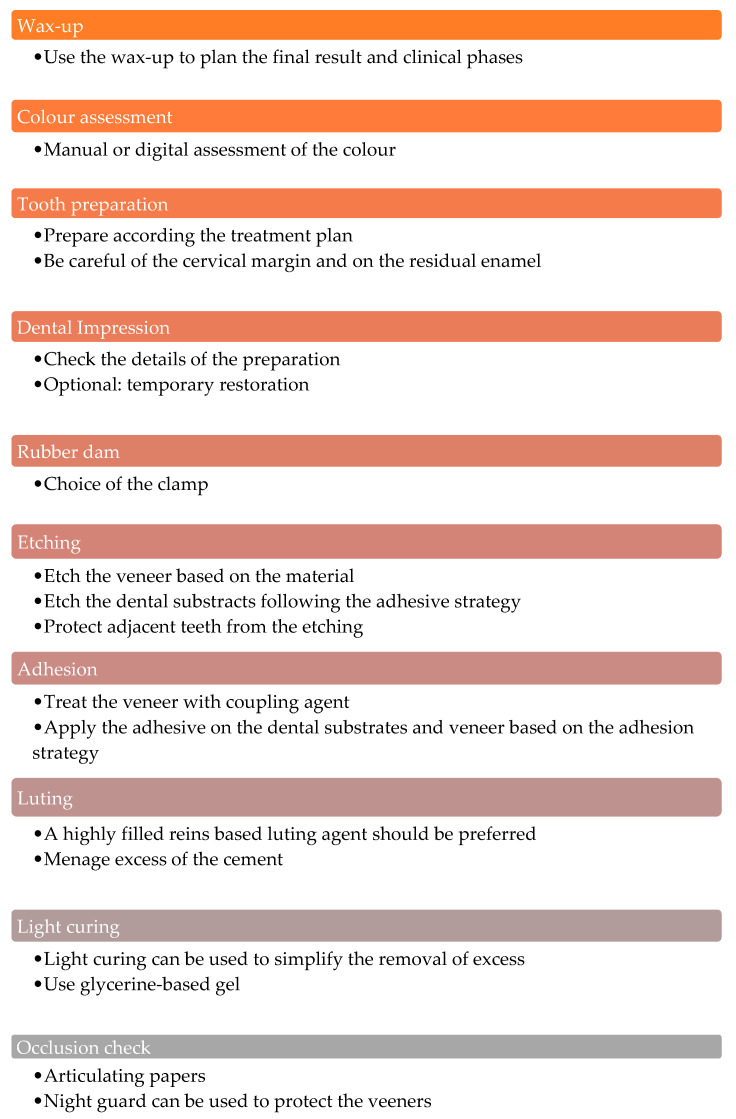
Indirect restoration with ceramic veneer phases.

Figure 5 summarizes the phases of performing an indirect restoration with ceramic veneers [25,26,27,28,29,30]. The operative phases and preparation types for indirect composite resin veneers are very similar to those for ceramic veneers, with some differences [28,29,31].

The enamel reduction for the tooth preparation can be lower if compared to ceramic veneers. During the clinical check before luting, the composite veneer can be modified if necessary [32]. The internal surface of composite resin veneers can be roughened with sandblasting or a coarse-grit diamond bur to obtain microretentions that increase the adhesion. After that, the veneer must be cleaned with alcohol, and the silane agent should be applied. After cleaning the tooth surface, the enamel has to be acid etched and rinsed with water, followed by the application of adhesive resin and air thinning.

The luting phase is the same as that for ceramic veneers. For the finishing of composite veneers, fine-grit diamond burs, silicone points, brushes impregnated with diamond paste, and aluminum oxide can be used.

In Table 5, we describe the advantages and disadvantages of the additive technique with indirect restoration, while Table 6 summarizes the indications and contraindications of this approach.

Nowadays, composite resin can be considered as an alternative to porcelain due to the excellent results they produce in the anterior sectors [28,29]. Ceramics can effectively reproduce the structure and translucency of the natural tooth. Dental ceramic are reinforced heterogeneous porcelains with a greater percentage of crystalline phase than porcelain fused to metal [28]. The use of resin composite for anterior indirect restorations is more recent than ceramics. The materials used are highly filled microparticle hybrid composites made up of glass filler (70% to 85% in weight) and particles varying from 0.04 to 3 μm in size [28]. If compared to ceramic veneers, composite veneers have shown important advantages, which could lead to the increase in their use in the future. Firstly, the phases of finishing and polishing are easier and less likely to increase fracture risk; furthermore, they can be modified before luting without compromising their mechanical or adhesive properties; finally, the cost is lower as laboratory procedures are simpler [28].

In an in vitro study conducted in 2020 [25], five preparation designs for ceramic laminate veneers were subjected to the forces of a chewing simulator and compared. They were as follows: non-prep, minimally invasive (no dentine exposed), semi-invasive (50% dentin exposed), invasive (labial preparation 100% in dentin) and the presence of two proximal class III resin composite restorations in the mid-third of the crown. The margin quality after the simulation appeared to be perfect for all the preparation designs. The fracture risk for thin veneers with marginal preparations in dentin was statistically higher if compared to veneers cemented only in enamel. Pre-existing class III composite resin restorations did not show an adverse effect on margin quality and fracture resistance. According to a study published in 2011 by Schmidt et al. [30], the preparation design and quantity of existing tooth structures significantly influence the load to failure of ceramic veneers. A greater exposure of transverse cross-sections of enamel prisms can increase resin–enamel bonding, as this is better for tooth preparation and acid etching on a transverse cross-section of enamel prisms than on a longitudinal cross-section. Furthermore, if the incisor tooth structure is reduced, the risk of adhesive bond failure increases, because of the decrease in enamel bonding. These finding are in accordance with another study published in 2011 [33], in which it was found that veneers prepared entirely on the enamel had superior fracture-strength values if compared with those prepared entirely on dentinal tissue. Moreover, the area of the enamel may also influence the bonding of resin-based composites, as the enamel on the vestibular surface may differ from that on the palatal/lingual surface [34].

The esthetic result of an anterior restoration also depends on the shape and color of the final restoration. The long-term success of anterior indirect restorations depends on the definition of adequate treatment planning to reduce the risk of failure [28]. For ceramic veneers, the literature reports satisfactory results in terms esthetic result, biocompatibility and periodontal response [28,35]. Clinical studies report survival rates over 90% at up to 4 to 10 years of follow-up; the main reasons for failures of this type of restoration are minor fractures of ceramics and marginal defects [29]. As regards indirect composite veneers, surface roughness, adaptation and marginal staining are the most frequent causes of failures. Over the long term, surface quality changes tend to be more frequent in indirect composite veneers; thus, they could require more maintenance but this drawback can be due to the physical characteristic of composite [29]. For anterior composite restorations, the literature reported survival rates at 5 years ranging from 85 to 89% [36,37]. A study published in 2012 [38] reported that neither the amount of residual tooth structure nor the amount of composite used affects fracture resistance. The main reason for the failure of the restoration is represented by the fracture of the composite resin, which in most cases occurs during the first year after the reconstruction of the dental element. The advantage of the relatively easy reparability of the resin composite is certainly a factor that extends the survival of composite restorations [39,40]. However, it is essential to consider the recent advancements in hybrid ceramics for 3D printing. These materials have the potential to change the frequency and clinical indications for the use of indirect composite restorations, owing to their enhanced properties and versatility compared to traditional options. 

While the prevention of MEF remain the preferred approach, when they are attributable to dental wear, it is important to correctly diagnose its etiology. If the diagnosis is bruxism, the causal therapy is psychological and physiotherapy, while a possible symptomatic therapy is wearing a night guard, which could limit dental wear [41]. A night guard is not useful when the cause of tooth wear is erosion; in this case, a treatment with fluoride could reinforce the tooth surface and increase its resistance to acid dissolution. Additionally, a review of the patient’s dietary habits, including an analysis of their food diary, should be conducted to identify and address potential dietary contributors [2]. Naturally, bad habits such as nail-biting should be avoided. 

## 4. Conclusions

This work summarizes different approaches to restoring teeth affected by MEFs. Selective the appropriate additive or subtractive technique can lead to adequate results both in aesthetic and functional terms, as well as the predictability of the result. The clinical approach needs to be both therapeutic and causal, because the control of etiological factors of MEF is fundamental for long-term prognosis. Restoring teeth affected by MEF requires a comprehensive evaluation that integrate an aesthetic, functional and causal considerations. Among the available techniques, the additive technique appears to be preferred in most cases, if compared to the subtractive technique, which presents limited indications. Furthermore, recent advancements in hybrid ceramics may reshape the decision-making process regarding the use of ceramic or composite for indirect veneers.

## Figures and Tables

**Table 1:** Some advantages and disadvantages of the subtractive technique.

Advantages	Disadvantages
Simple and quick execution Economic Possibility of use in moments of transition For patient who prefers a fast and economical treatment	Not conservative Not retractable Possible esthetic limits: morphological changes can oblige to intervene on the healthy contralateral element

**Table 2:** Some indications and contraindications of the subtractive technique.

Indications	Contraindications
Before to perform small restorations that may present a retention problem Difference in height on the vertical plane of an element in comparison to the contralateral one in the case of short crowns Occlusal precontacts and in lateral and protrusive movements Severe deep bite, inability to increase the tooth length Transformation of the canine into a lateral incisor with direct composite camouflage (canine substitution technique) To reshape a tooth that appear morphologically unpleasant for the patient Orthodontic finishing	Tooth with short crown

**Table 3:** Some advantages and disadvantages of the direct composite restoration.

Advantages	Disadvantages
Acceptable longevity More aesthetic than the subtractive technique, the original shape of the tooth can be restored To achieve correct form and function, with anatomical and functional restoration Faster than the indirect technique More conservative than the indirect technique, minimal preparation Cheaper than the indirect technique Possibility of repairs and subsequent replacements of the restoration simplified if compared to the indirect technique, without the need to remake the whole restoration	Possible detachment of the restoration Aesthetic problem of the restoration over time due to discoloration

**Table 4:** Some indications and contraindications of the direct composite restoration.

Indications	Contraindications
Young patients Waiting situations (based on biological and/or strategic criteria) Reconstruction after small tooth injuries	Large lesions, with low biomechanical resistance of the tooth to be restored

**Table 5:** Advantages and disadvantages of the indirect restoration.

Advantages	Disadvantages
Aesthetics Greater resistance to wear Biocompatibility with soft tissues Dimensional and chromatic stability over time Strong bond between the two adhesive interfaces	Costs More time than the direct technique (more sessions) Less conservative than the direct technique, more extensive tooth preparation For ceramic veneers, greater wear on the enamel of the antagonist teeth

**Table 6:** Indications and contraindications of the indirect restoration.

Indications	Contraindications
Patients with complex therapeutic goals, major rehabilitations Large lesions, with low biomechanical resistance of the tooth to be restored Difficulty to obtain a satisfactory aesthetic result for the patient (for example, in the case of a dyschromic devitalized tooth) Aesthetic needs of the patient Failure of the direct technique	Patient with no complex therapeutic objectives Small restorations Adolescents and growing patients

## Data Availability

Data and information about the paper will be provided upon request.
